# Prospective Observational Case-Control Study on the Potential of Maternal Ophthalmic Artery Doppler Velocimetry in the Evaluation of Preeclampsia

**DOI:** 10.7759/cureus.81983

**Published:** 2025-04-09

**Authors:** Sharanabasava S Kulkarni, Shreedevi Kori, Satish D Patil, Rajasri G Yaliwal, Subhashchandra Mudanur, Shobha Shiragur, Aruna Biradar

**Affiliations:** 1 Department of Obstetrics and Gynaecology, BLDE (Deemed to Be University) Shri B.M. Patil Medical College, Vijayapura, IND; 2 Department of Radiodiagnosis, BLDE (Deemed to Be University) Shri B.M. Patil Medical College, Vijayapura, IND

**Keywords:** high-resolution ultrasound, maternal morbidity, opthalmic artery doppler, preeclampsia, pregnancy-induced hypertension (pih)

## Abstract

Background and objective

Preeclampsia (PE) is a lethal hypertensive disorder that significantly contributes to maternal as well as fetal morbidity besides mortality, especially in low-resource settings like India. Thus, it is essential to diagnose and manage early to prevent poor maternal and fetal outcomes. PE causes cerebrovascular endothelial dysfunction and impairs cerebral autoregulation due to systemic hypertension. So, monitoring of cerebrovascular alteration is crucial for preventing severe neurological outcomes. In this study, we have used maternal ophthalmic artery Doppler (OAD) as a key tool for monitoring hemodynamic parameters and severity of the disease in PE and normotensive pregnant women.

Methodology

A prospective observational case-control study took place in a medical facility that provided tertiary care from April 2023 to March 2025, including 170 pregnant women (85 PE cases and 85 normotensive controls). OAD metrics, such as pulsatility index (PI), resistivity index (RI), peak systolic velocity, and end-diastolic velocity, were measured using high-resolution ultrasound with a 7-10MHz transducer. Data were analyzed using IBM SPSS Statistics for Windows, Version 20 (Released 2011; IBM Corp., Armonk, New York, United States), and cutoff values for determining PE severity were determined using receiver operating characteristic (ROC) curve analysis.

Results

PE patients demonstrated significantly higher RI and PI values compared to controls (p<0.001). ROC analysis identified RI >0.72 and a strong predictor of PE severity (sensitivity 82.3%, specificity 79.4%). Increased OAD indices correlated with adverse maternal and fetal consequences, including intrauterine growth restriction as well as preterm birth.

Conclusion

This prospective observational case-control study demonstrates the maternal OAD velocimetry as a valuable tool in evaluating and predicting PE in pregnancy. Increased resistance in the right ophthalmic artery and lower pulsatility suggest cerebrovascular dysfunction and impaired autoregulation in PE. As it serves as a promising surrogate marker for cerebrovascular dysfunction, its integration into routine obstetric evaluation may aid in early detection and risk stratification.

## Introduction

Preeclampsia (PE) is a disease that appears after 20 weeks of gestation and may lead to eclampsia, contributing to high maternal mortality besides maternal morbidity, particularly in resource-limited areas like India. Pregnancy-induced hypertension (PIH) is a key factor in maternal mortality, emphasizing the need for better diagnostic and management strategies. Neurological complications, such as stroke and visual disturbances, arise from cerebrovascular dysfunction due to high blood pressure (BP). However, real-time monitoring using CT or MRI is limited by safety, cost, and logistical challenges, especially in obstetric care [1-3].

Transcranial Doppler ultrasonography has a limited role in examining large-caliber intracranial arteries, like the middle cerebral artery, despite being a real-time, non-invasive method of assessing cerebral hemodynamics. Such arteries may fail to represent pathophysiological and microvascular alterations that take place in PE. Consequently, there is a growing interest in exploring alternative vascular territories that can serve as surrogates for cerebrovascular evaluation in PE [4-6].

Because of its origin, anatomy, and function correlated with that of the brain's blood supply, the ophthalmic artery is noteworthy. The analysis of vascular alterations in glaucoma, multiple sclerosis, systemic atherosclerosis, and heart failure has extensively used Doppler ultrasonography scanning of the ocular artery. Its relatively low cost, non-invasiveness, and capacity to provide real-time hemodynamic data suggest that it may be invaluable for obstetric purposes [7-12]. While several studies have explored cerebral hemodynamics in PE, the role of ophthalmic artery Doppler (OAD) remains underexplored. This study aims to bridge this gap by evaluating its utility as a non-invasive cerebrovascular marker in PE.

This intended case-control study intends to fill this gap by investigating maternal OAD velocimetry as a possible indicator of cerebrovascular hemodynamics in cases of PE. With this form of imaging at hand, we hope to develop a simple and inexpensive approach to evaluate changes in the cerebrovascular system of the patients, which would be beneficial in the management as well as estimation of PE outcomes. This investigation not only highlights the potential of OAD velocimetry in obstetric care but also sets the stage for its integration into routine clinical practice for early discovery plus management of PE.

## Materials and methods

A prospective observational case-control study was conducted at BLDE (DU) Shri B.M. Patil Medical College and Hospital and Research Centre, Vijayapura, Karnataka, India, from April 2023 to March 2025. Ethical clearance was obtained (IEC/897/2022-23), and the study is registered in the clinical trial registry of India (CTRI/2023/09/057600). The purpose of these conditions was to guarantee a uniform profile of patients for the assessment process.

**Figure 1:**
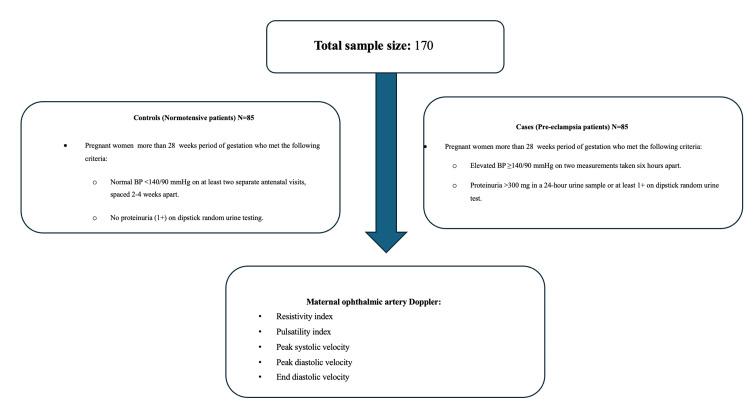
Case and control distribution.

This study included 170 pregnant women, 85 PE cases, and 85 normotensive controls for more than 28 weeks of gestation. Participants with a history of smoking, substance abuse, diabetes, chronic hypertension, ocular pathology, corticosteroid use, antihypertensive therapy, or vascular conditions affecting Doppler measurements were excluded from the study. A comprehensive physical examination and medical history were conducted. A 7-10 MHz transducer was utilized in high-resolution ultrasonography to evaluate OAD parameters such as RI, PI, and end-diastolic velocity (EDV), along with peak systolic velocity (PSV) (Figure 1).

Case group

The PE group consisted of pregnant women over 28 weeks with elevated blood pressure (BP ≥140/90 mmHg) on two successive measurements taken minimum difference of six hours and proteinuria over 300 mg in a 24-hour urine sample or minimum 1+ on a dipstick random urine testing. 

Control group

The control group consists of pregnant women within the same gestational age with normal blood pressure (BP <140/90 mmHg) included if there was no proteinuria (1+ on dipstick random urine testing) and at least two prenatal visits separated by 2-4 weeks (Figure 1).

**Figure 2:**
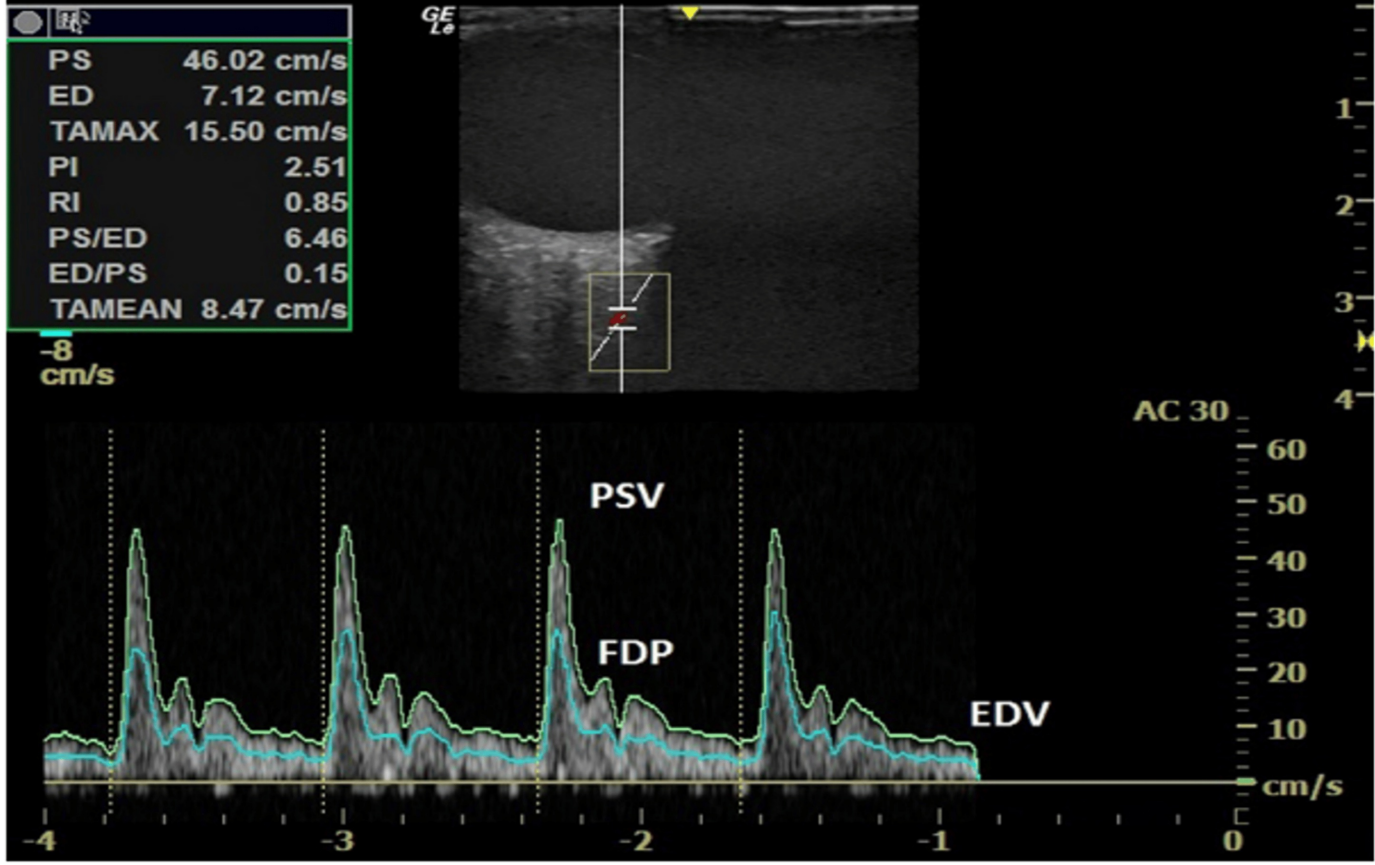
B-mode ultrasound image of orbit and retro-orbital structures, with superimposed color Doppler for identification of the ophthalmic artery, and pulsed-wave Doppler to acquire its spectral waveform. EDV: End-diastolic velocity; FDP: first diastolic peak velocity; PSV: peak systolic velocity.

In both groups, OAD velocimetry was performed using the following method. An individual was put in a supine pose with minor left lateral rotation. Using a GE VOLUSON S8 BT18 ultrasound machine (WIPRO GE Healthcare, Bangalore, India) with a 50/60Hz linear transducer, the ophthalmic artery was located via a color Doppler flow image. Following 10 minutes of relaxation, the upper eyelid was lightly coated with acoustic gel. The right ophthalmic artery was insonated first, followed by the left, with flow velocity assessed around 15 mm from the optic disc. Key Doppler parameters, including PDV, PSV, EDV, PI, and RI, were all measured (Figure 2).

Statistical analysis

Microsoft Excel was utilized to handle data, while IBM SPSS Statistics for Windows, Version 20 (Released 2011; IBM Corp., Armonk, New York, United States) was utilized for statistical analysis. Results are shown as diagrams, counts, percentages, and mean ± SD. For continuous variables amongst these two groups, the independent t-test was employed; for non-normally distributed data, the Mann-Whitney U test was employed. The category variables of these two groups were matched through the chi-square test. The cutoff values for pertinent OAD parameters were determined using the receiver operating characteristic (ROC) curve. All tests employed a two-tailed methodology, with a p-value of <0.05 considered statistically substantial.

## Results

The patients with normotension were enlisted after 28 weeks of gestational age. Of the mothers, 40% were between the ages of 30 and 34. The majority of the female patients were multiparous. Their systolic BP ranged between 110 and 119 mmHg (40%), and their diastolic BP ranged between 70 and 79 mmHg (44.7%) (Table 1).

**Table 1:** Demographic characteristics of the normal population (controls). BP: Blood pressure; GA: gestational age

Characteristics	Frequency (n=85)	Percentage
Age in years		
<30	20	23.5
30-34	34	40
35-39	21	24.7
≥40	10	11.8
GA (weeks)		
<30	0	0
30-34	6	7
35-39	54	63.5
≥40	25	29.4
Parity		
0	24	28.2
1	35	41.2
2	18	21.2
≥3	8	9.4
Systolic BP (mmHg)		
<100	9	10.6
100-109	15	17.6
110-119	34	40
≥120	27	31.7
Diastolic BP (mmHg)		
<60	0	0
60-69	25	29.4
70-79	38	44.7
≥80	22	25.9

Most PE patients (63%) appeared between gestational age weeks 30 and 34. The majority of them were nulliparous and often under 30 years old. As indicated in Table 2, their systolic and diastolic BP were most frequently between 160 and 179 mmHg (54.1%) and 90 and 109 mmHg (58.8%), respectively

**Table 2:** Demographic characteristics of cases. BP: Blood pressure; GA: gestational age

Characteristics	Frequency (n=85)	%
Age in years		
<30	30	35.3
30-34	28	33
35-39	20	23.5
≥40	7	8.2
GA (weeks)		
<30	0	0
30-34	45	53
35-39	30	35.3
≥40	10	11.7
Parity		
0	30	35.3
1	25	29.4
2	10	11.8
≥3	20	23.5
Systolic BP (mm Hg)		
140-159	15	17.6
160-179	46	54.1
180-199	19	22.3
≥200	5	5.9
Diastolic BP (mm Hg)		
90-109	50	58.8
110-119	15	17.6
120-139	16	18.8
≥140	4	4.7

The mean values of OAD parameters were compared among pre-eclamptic as well as control groups. The pre-eclamptic group had a greater right RI (0.73 ± 0.54) than the control group (0.71 ± 0.21), with a p-value of 0.00 suggesting statistical importance. The pre-eclamptic group’s left RI was also considerably lower (0.69 ± 0.65), matched with the control group's RI (0.71 ± 0.12, p = 0.01). For the PI, both right and left PI values were considerably lower in the pre-eclamptic group (1.3 ± 0.62 and 1.24 ± 0.45) than in the control group (1.78 ± 0.56 and 1.58 ± 0.53), with p-values of 0.00. 

Regarding PSV, the right and left PSV readings were lower in the pre-eclamptic group (29.25 ± 4.45 cm/sec and 29.76 ± 4.67 cm/sec) compared to the control group (32.10 ± 3.45 cm/sec and 34.02 ± 4.78 cm/sec), However, the differences (respectively, p = 0.16 and p = 0.18) were statistically insignificant. For PDV, the right PDV was considerably higher in individuals who were pre-eclamptic cases (23.10 ± 2.45 cm/sec) than in the control group (19.87 ± 2.85 cm/sec, p = 0.04). Similarly, the left PDV was notably greater in the preeclamptic group (25.67 ± 3.95 cm/sec) than in the control group (20.45 ± 3.56 cm/sec, p = 0.00). Finally, EDV values were slightly higher in the pre-eclamptic group for both right (11.02 ± 3.56 cm/sec) and left (11.67 ± 3.54 cm/sec) sides, compared to the control group (8.23 ± 2.67 cm/sec and 7.98 ± 3.12 cm/sec). The statistical significance was also observed for the left EDV (p = 0.05) as well as the right EDV (p = 0.04) (Table 3).

**Table 3:** Comparison of OAD parameter values related to preeclampsia and control groups. RI: Resistivity index; PI: pulsitivity index; EDV: end-diastolic velocity; PSV: peak systolic velocity; PDV: peak diastolic velocity

Variables (cm/sec)	Pre-eclamptic group Mean±SD	Control group Mean±SD	T-test	p-value
Left PSV	29.76±4.67	34.02±4.78	-1.85	0.18
Right PSV	29.25±4.45	32.10±3.45	-2.02	0.16
Left PI	1.24±0.45	1.58±0.53	-4.56	0.00
Right PI	1.3±0.62	1.78±0.56	-3.43	0.00
Left RI	0.69±0.65	0.71±0.12	-3.89	0.01
Right RI	0.73±0.54	0.71±0.21	-4.43	0.00
Left EDV	11.67±3.54	7.98±3.12	3.32	0.05
Right EDV	11.02±3.56	8.23±2.67	3.46	0.04
Left PDV	25.67±3.95	20.45±3.56	3.89	0.00
Right PDV	23.10±2.45	19.87±2.85	-2.37	0.04

Comparison of BP and OAD metrics among individuals with severe or mild PE

The comparison of BP and OAD parameters among individuals with severe or mild PE revealed significant differences in certain variables.

Blood Pressure

Patients with severe PE had substantially greater systolic BP (179.34 ± 22.53 mmHg) than mild PE patients (148.78 ± 8.65 mmHg, p = 0.00). Similarly, diastolic BP was significantly elevated in the severe PE group (110 ± 15.48 mmHg) compared to the mild PE group (98.23 ± 5.08 mmHg, p = 0.02).

OAD Parameters

In comparison to the mild PE group (0.71 ± 0.06, p = 0.01), the severe PE group (0.62 ± 0.18) had a considerably lower right RI. Similarly, the right PI was significantly reduced in severe cases (1.06 ± 0.37) compared to mild PE (1.27 ± 0.25, p = 0.03), suggesting altered vascular resistance in severe cases (Table 4).

**Table 4:** Comparison of blood pressure and ophthalmic artery Doppler metrics relating to individuals with severe or mild preeclampsia PE: Preeclampsia; PI: pulsatility index; RI: resistive index; EDV: end diastolic velocity; PDV: peak diastolic velocity; SBP: systolic blood pressure; DBP: diastolic blood pressure; PSV: peak systolic velocity

Variables	Mild PE (n=46) Mean±SD	Severe PE (n=39) Mean±SD	t value	p-value
Right EDV (cm/sec)	9.73±1.86	10.41±1.39	0.98	0.57
Right PDV (cm/sec)	22.39±1.98	22.46±2.04	0.87	0.88
Right PSV (cm/sec)	29.55±3.16	26.80±3.66	-0.44	0.78
Right PI	1.27±0.25	1.06±0.37	-2.89	0.03
Right RI	0.71±0.06	0.62±0.18	-2.98	0.01
SBP (mmHg)	148.78±8.65	179.34±12.53	5.67	0
DBP (mmHg)	98.23±5.08	110±5.48	6.45	0.02

Other OAD parameters, including the right EDV, right PDV, and right PSV, displayed no noteworthy contrasts among groups (p = 0.057, 0.88, 0.78 respectively), representing that while vascular resistance was affected, overall blood flow velocities did not significantly differ.

## Discussion

This prospective observational case-control study aimed to evaluate the association between maternal OAD parameters and PE, leveraging this non-invasive imaging modality to assess cerebrovascular hemodynamics. The study's findings demonstrate significant alterations in OAD indices between preeclamptic and normotensive pregnant women, as well as differences between severe and mild PE cases, underscoring the potential of this technique in managing this condition.

Relationship with earlier research

Our findings align with those of Olatunji et al. [13], who observed a significant reduction in the RI and PI among preeclamptic patients. However, unlike Olatunji et al., our study reported a higher right RI in PE cases, suggesting potential regional variability. Olatunji et al. [13] observed significantly lower RI, PI, and PSV in pre-eclamptic individuals compared to normotensive controls, similar to the reductions seen in our study. Additionally, the higher PDV and EDV in the preeclamptic group align with the conclusions of Barbosa et al. [14], who reported increased downstream resistance and altered cerebrovascular hemodynamics in severe PE.

However, our study found a higher right RI in the preeclamptic group, which is in contrast to some earlier studies that reported lower RI values in PE [14]. This inconsistency may be attributed to variances in patient populations, disease severity, or regional variations in the vascular response to hypertension. The reduced right and left PI in our preeclamptic participants also contradicts the findings of Olatunji et al. [13], who observed higher PI values in their study cohort.

Strengths and limitations

One key strength of this investigation is its prospective observational case-control design, which enabled a direct judgment of OAD parameters among normotensive and preeclamptic pregnant women. The employment of a standardized Doppler assessment protocol, as outlined by Lieb et al. [15], provided consistency in data collection and reduced potential measurement biases. The study's emphasis on both mild and severe PE cases also offers important insights into the correlation between OAD indices and disease severity.

A limitation of this study is its single-center design, which may restrict generalizability. A small sample size, although sufficient to detect group differences, might have compromised the statistical power of some analyses, especially when comparing between mild and severe PE subgroups. Lack of follow-up along the longitudinal dimension also inhibits testing of postpartum alterations in Doppler parameters and their relationship with long-term neonatal and maternal outcomes.

Further studies are needed to validate the diagnostic and prognostic value of OAD in PE, ideally by larger, multicenter trials with extended follow-up. The inclusion of other clinical and biochemical markers, including uterine artery Doppler and angiogenic factors, may also increase the predictive value of this non-invasive imaging modality.

Clinical implications

The current findings highlight the promise of maternal OAD as a useful tool for evaluating cerebrovascular alterations in PE. The noted changes in RI, PI, PDV, and EDV indicate that this point-of-care imaging technique has the potential to offer helpful information on the pathophysiology of the condition and may assist in early detection, risk stratification, and management. The potential to discriminate between mild or severe PE, as per Doppler parameters, could assist clinicians in personalizing interventions and enhancing maternal and fetal outcomes.

Utilizing the accessibility, affordability, and real-time advantages of OAD, the method could be incorporated into day-to-day obstetric practice, especially in under-resourced regions where high-technology imaging tools might not be readily available. Early detection of high-risk cases by Doppler examination can help initiate the timely implementation of preventive interventions and vigilant surveillance, thus preventing the development of PE-associated complications.

## Conclusions

This prospective observational case-control study demonstrates that maternal OAD velocimetry is a valuable means of evaluating and predicting PE in pregnancy. Increased resistance in the right ophthalmic artery and lower pulsatility suggest cerebrovascular dysfunction and impaired autoregulation in PE. By taking advantage of the accessibility, affordability, and real-time nature of OAD, this method can be incorporated into standard obstetric practice, especially in resource-limited settings where sophisticated imaging modalities are not readily available. Early detection of high-risk patients by OAD evaluation can facilitate the early application of preventive measures and close follow-up, thereby decreasing the burden of PE-related complications.
